# How flood risks shape policies: flood exposure and risk perception in Swiss municipalities

**DOI:** 10.1007/s10113-020-01705-7

**Published:** 2020-10-11

**Authors:** Anik Glaus, Markus Mosimann, Veronika Röthlisberger, Karin Ingold

**Affiliations:** 1 Institute of Political Science, University of Bern, Fabrikstrasse 8, 3012 Bern, Switzerland; 2 Oeschger Centre for Climate Change Research, University of Bern, Hochschulstrasse 4, 3012 Bern, Switzerland; 3 Institute of Geography, University of Bern, Hallerstrasse 12, 3012 Bern, Switzerland; 4 Mobiliar Lab for Natural Risks, University of Bern, Hallerstrasse 12, 3012 Bern, Switzerland; 5 Eawag, Swiss Federal Institute for Aquatic Science and Technology, Überlandstrasse 133, 8600 Dübendorf, Switzerland

**Keywords:** Flooding, Flood risk management, Risk perception, Policy preferences, Policy design

## Abstract

Despite an increasing number of people exposed to flood risks in Europe, flood risk perception remains low and effective flood risk management policies are rarely implemented. It becomes increasingly important to understand how local governments can design effective flood risk management policies to address flood risks. In this article, we study whether high flood exposure and flood risk perception correlate with the demand for a specific design of flood risk management policies. We take the ideal case of Switzerland and analyze flood risk management portfolios in 18 flood-prone municipalities along the Aare River. We introduce a novel combination of risk analysis and public policy data: we analyze correlations between recorded flood exposure data and survey data on flood risk perception and policy preferences for selected flood risk management measures. Our results indicate that local governments with high flood risk perception tend to prefer non-structural measures, such as spatial planning and ecological river restoration, to infrastructure measures. In contrast, flood exposure is neither linked to flood risk perception nor to policy preferences. We conclude that flood risk perception is key: it can decisively affect local governments’ preferences to implement specific diversified policy portfolios including more preventive or integrated flood risk management measures. These findings imply that local governments in flood-prone areas should invest in raising their population’s awareness capacity of flood risks and keep it high during periods without flooding.

## Introduction

A growing number of extreme flood events in Europe poses an increasing risk to people, assets, and infrastructure (Kundzewicz et al. 2018b). Damages and losses caused by floods are high and constitute a financial burden for numerous European economies (Kron et al. 2019). However, increasing flood risk is not only due to changing climate conditions or human development in flood-prone areas (Löschner et al. 2017) but also to a lack of flood preparedness (Kundzewicz et al. 2020) and effective flood risk management (Intergovernmental Panel on Climate Change (IPCC) 2007).

Although more and more people are exposed to increasing flood risks (Nicholls et al. 2008; Kundzewicz et al. 2014), their perception of flood risks does not correspond with their actual exposure (Botzen et al. 2009). Public defense investments and technological advances often provide exposed people with a false sense of safety (Baron and Petersen 2015; Kellens et al. 2011; Kron et al. 2019). The underestimation of flood risks reduces public support for policies and the willingness to take preventive measures (Botzen et al. 2009). Therefore, in this article, we are interested in how local governments can devise an effective portfolio of policies to address flood risks. We argue that effective flood risk management is possible when people’s exposure to floods, their perception of flood risks, and their policy preferences to address flood risks are congruent. In other words, if flood exposure, flood risk perception, and policy preferences diverge, it is difficult to introduce effective flood risk management measures.

Continuous urban developments in many flood-prone areas increase people’s exposure to floods (Kron et al. 2019) and, thus, put pressure on local governments to invest in flood risk management. Nevertheless, citizens’ lack of problem perception undermines local governments’ legitimacy to implement effective policies (Botzen et al. 2009). The apparent mismatch of flood exposure, flood risk perception, and policy preferences is surprising and poses a serious challenge for flood risk management. To our knowledge, there is no empirical research on the interplay between these three variables. In this context, our primary research question is: *To what extent are flood exposure, flood risk perception, and policy preferences related?* To also better understand policy preferences in flood-prone areas, we further ask: *Which flood risk management measures do local governments prefer in a flood-prone catchment area?*

**Fig. 1:**
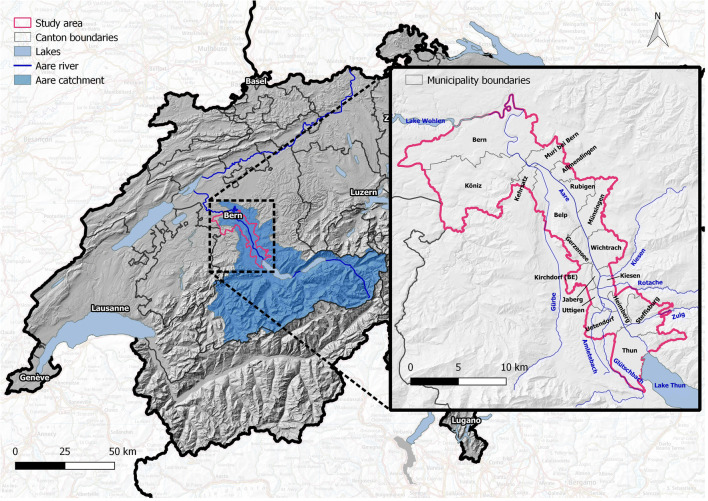
Sub-catchment area of the Aare River with its 18 municipalities

Empirically, we focus on the case of Switzerland with its long-standing experience and expertise in natural disaster risk reduction. We study flood risk management portfolios in 18 flood-prone Swiss municipalities in the hydrological sub-catchment area of the Aare River between the cities of Thun and Bern in the Canton Bern (Fig. 1 in Appendix 1). Due to its geographic position at the source of several large European rivers, combined with its small size and dense settlement, Switzerland has a long tradition in flood risk management (Ingold and Gavilano 2020), for which Swiss cantons, together with the federal and municipal governments, are responsible. As such, a wide range of policies exists within the 26 Swiss cantons and their municipalities, which address floods and other natural hazards. Switzerland therefore proves an ideal example for learning from past experiences for today’s design of flood risk management policies.

Our article adds the value of a novel combination of risk analysis and public policy data: first, we consider flood exposure data from recorded floodings between 1997 and 2016 and georeference it to affected buildings and residents in the municipalities in our sub-catchment area; second, we survey local governments’ representatives on flood risk perception and policy preferences for selected measures in the 18 municipalities in our sub-catchment area; third, we analyze the interplay of the 18 municipalities’ flood exposure, flood risk perception, and policy preferences with Spearman’s rank-order correlations and Cronbach’s alpha. By understanding the link between these three variables, we aim to shed light on mechanisms influencing the design of effective local flood risk management portfolios and learn from regional flood risk management to effectively reduce flood risks.

## Context of the three key variables

### Flood exposure

Following the UN terminology, flood exposure can be defined as “the people, property, systems, or other elements present in flood zones that are thereby subject to losses” (United Nations Office for Disaster Risk Reduction (UNISDR) 2009). Exposure is understood as one of the three key components of the risk function, besides hazard—the occurrence probability of an extreme event, and vulnerability—a society’s capacity to deal with an extreme event (Intergovernmental Panel on Climate Change (IPCC) 2012, Kron et al. 2019). Increasing risk is usually the result of increasing exposure and vulnerability, hence non-climatic mechanisms that can be attributed to human activities in flood-prone areas (Kundzewicz et al. 2020). Thus, flood risk management policies often concentrate on the reduction of a society’s exposure and vulnerability to flood risks (Koks et al. 2015). In this article, we focus on flood exposure because continuing population growth in flood-prone areas by simultaneous ignorance of flood risks is a relevant issue (Kundzewicz et al. 2014).

It is important to differentiate between recorded (or occurred) exposure and modelled (or potential) exposure. Modelled exposure refers to those elements (e.g., buildings or residents) that are potentially affected by floods, according to the hypothetical flood scenarios calculated with numeric models. By contrast, recorded exposure relates to those elements that were affected by flood events in the past, i.e., which were located in the flooded areas. In this article, we focus on recorded exposure, since flood risk management builds on what happened in the (immediate) past rather than on what could potentially occur in the future (Suter et al. 2016).

### Flood risk perception

In the literature, risk perception is defined as the combination of individual judgments of a hazard’s probability with the perceived severity of potential consequences (Griffin et al. 2008). In addition to this rational and analytical definition (*risk as analysis*), risk perception also includes an affective component (*risk as feelings*) (Slovic et al. 2004). Thus, the study of flood risk perception also concerns people’s awareness, emotions, and behavior related to flood risks (Kellens et al. 2011). Flood risk judgments can vary between individuals and groups in the same catchment area because they hold different information on flood risks, unequal levels of uncertainty, specific political power constellations, or particular interests (Slovic 1987). Although there is a general awareness of high flood damages in flood-prone areas, people hardly heed the potential consequences of flooding. To increase people’s perception, the public discourse on climate change and extreme events tends to be negatively framed in terms of risk, damage, and fear (Kundzewicz et al. 2020), a phenomenon called “atmosfear” (Janković and Schultz 2017). However, as the literature states, rather than scaring people, enhancing their knowledge on flood risks (e.g., by explaining multiple mechanisms causing floods and how these mechanisms are linked to human activities) (Kundzewicz et al. 2020) and including them in flood processes via participatory approaches (Driessen et al. 2018) contribute to people’s flood risk perception and their acceptance of and support for flood risk management policies (Otto-Banaszak et al. 2011; Otto et al. 2020).

### Policy preferences

Policy preferences reflect political actors’^1^ level of support or opposition to proposed policies and measures in different policy fields (Leiserowitz 2006). Therefore, policy preferences constitute an important precondition for the successful implementation of policies and measures (Dermont et al. 2017).

In terms of political actors’ preferences for effective flood risk management policies, recent literature suggests a diversified mix of strategies and measures tailored to the country-specific context (i.e., physical and geographical conditions, historical flood risk management, societal and cultural norms, administrative and legal frameworks) (see STAR-FLOOD project publications, e.g., Driessen et al. 2018, Driessen et al. 2016, Hegger et al. 2014, Hegger et al. 2016, Kundzewicz et al. 2018a). However, in European flood risk management, infrastructure (e.g., dams) remains the primary, most visible, and widespread category of measures (Gralepois et al. 2016), since it deploys immediate effectiveness in case of flooding. Infrastructure stands in contrast to the less popular, but more diversified non-structural categories of measures such as spatial planning (e.g., construction bans), ecological river restoration (e.g., riverbed widening), and information tools (e.g., warning systems). Non-structural measures take longer to implement than infrastructure measures and influence flood impacts rather indirectly. In summary, many European countries show strong preferences for flood defense (i.e., infrastructure); however, an emerging tendency towards broadened preferences for more diversified strategies such as flood prevention (i.e., spatial planning), flood preparation (i.e., information), or flood mitigation (i.e., more natural measures) can be observed (Hegger et al. 2016).

### Relation between key variables

The aim of our research is to investigate the link between the three key variables outlined above. In a policy design process, problem affectedness and problem perception influence actors’ preferences for measures to solve the problem. Studying these policy preferences is essential for understanding local governments’ designs of flood risk management (i.e., the choice, implementation, and evaluation of policies and measures). Ostrom (2000) and Gerber et al. (2009) substantiate this relationship in their studies on the management of natural common-pool resources and institutional resource regimes: If actors are heavily dependent on a resource and its uses (e.g., farmers on arable land), their perception of potential threats to that resource (e.g., flooding) is high as they are directly affected. The more significant the potential threat to the resource, the more the affected actors will perceive the threat as a collective problem to be solved. High affectedness and strong perception lead to actors’ preferences and efforts to implement effective regulations and measures to protect the resource and its uses.

Several studies argue that actors’ experiences with past flood events influence their perception of flood risks (e.g., Wachinger et al. 2013). Other studies hypothesize that actors’ geographical proximity to a hazard source is a determinant of their flood risk perception (e.g., O'Neill et al. 2016). Thus, different degrees of flood exposure may lead actors to perceive flood risks differently. Deduced from that, our first hypothesis reads as follows:

- *Hypothesis 1: The higher the local governments’ exposure to flood risks, the stronger their perception of flood risks.*

On the other hand, literature indicates an influence of flood risk perception on actors’ flood preparedness and willingness to invest in private measures (Bubeck et al. 2012). However, this link is controversial (e.g., Miceli et al. 2008). Nevertheless, there exists some evidence that the degrees of flood exposure and flood risk perception affect actors’ preferred design of flood risk management (Messner and Meyer 2005). Actors’ direct affectedness through experienced exposure, combined with their strong perception of flood events, influences their support for or opposition to specific flood risk management measures (Leiserowitz 2006). Consequently, we might expect that highly exposed and flood-aware actors express different policy preferences than less exposed actors who perceive a lower flood risk (Tanner and Árvai 2018). Studying actors’ preferences for specific measures, we consider the concept of path dependency, which theorizes that actors tend to behave conservatively and defend existing patterns of policies. Actors’ policy preferences depend on formerly adopted policies and traditional policy instruments with well-known functioning and outcomes (Peters et al. 2005; Pierson 2000), which signifies a simple and reliable way to address flood risks and reduce uncertainty related to the accurate measures (Howlett 2005). Therefore, we expect actors to prefer the widespread and well-known infrastructure measures in contrast to the less popular categories of spatial planning, ecological river restoration, and information (Driessen et al. 2018; Otto-Banaszak et al. 2011). Deduced from that, our hypotheses 2 and 3 read as follows:

- *Hypothesis 2: The higher the local governments’ exposure to flood risks, the stronger their preferences for infrastructure measures to address flood risks.*
- *Hypothesis 3: The higher the local governments’ perception of flood risks, the stronger their preferences for infrastructure measures to address flood risks*.

## Materials and methods

### Case study: Swiss flood risk management and the sub-catchment area of the Aare River

Switzerland has a long history of flood risks due to its mountain regions acting as the source of several large European rivers paired with the country’s small size and its dense settlement (Ingold and Gavilano 2020). Swiss flood risk management is state-oriented and relies on public spending. According to the Federal Hydraulic Engineering Act (Federal Act of 21 June 1991 on Hydraulic Engineering), Swiss cantons (states) are responsible for flood risk management, while municipalities implement flood risk management strategies and measures. The federal government influences flood risk management by granting the cantons and municipalities compensation and subsidies. Consequently, many different flood risk management policies exist within the 26 cantons and their municipalities. Traditionally, Swiss flood risk management is characterized by a construction-oriented regime. However, a paradigm shift has taken place towards spatial planning-oriented approaches since the 1990s and integrated risk management since 2010 (Zaugg Stern 2006). This development corresponds to Article 3 of the Federal Hydraulic Engineering Act (Federal Act of 21 June 1991 on Hydraulic Engineering) establishing spatial planning and water body maintenance as the top priorities of Swiss flood risk management.

**Fig. 2:**
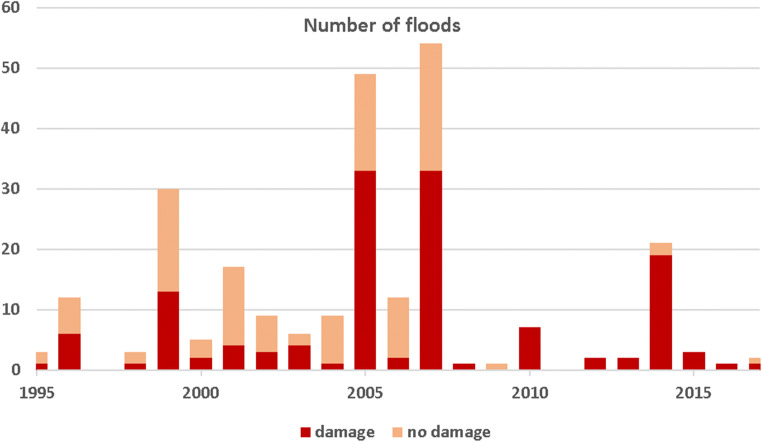
Historical flood records in the sub-catchment area of the Aare River from 1995 to 2017

In our case study, we focus on flood risk management in 18 flood-prone Swiss municipalities between the cities of Thun and Bern (Canton Bern) in a sub-catchment area of the Aare, one of the major rivers in Switzerland (Fig. 1 in Appendix 1). This case study region proves ideal for two reasons. First, this densely populated region has experienced several flood events during the last two decades. Three major flood events of May 1999, August 2005, and July/August 2007 led to significant damage on buildings and infrastructure along the Aare River and its tributaries. In the Canton Bern, the flood of 2005 caused a total damage of CHF 805 million (Bezzola and Hegg 2007). The maximum discharge values of the Aare River measured in Bern during the three mentioned events comply with the three highest values measured since the installation of the gauging station in 1918. They correspond to a statistical return period of more than 150 years (Federal Office for the Environment (FOEN) 2020). Besides these three major events, local minor events causing damage in the municipal area of this study were recorded for almost every year since 1995 (see Fig. 2 in Appendix 1; Forest and Natural Hazard Office Canton Bern (KAWA) 2018). Second, an action plan with defined measures is available within the selected sub-catchment area. We are therefore able to measure local governments’ exposure to floods, their perception of flood risks, and their policy preferences for various flood risk management measures.

### Survey data

We collected data in the sub-catchment area between December 2016 and January 2017 using a survey. First, we conducted 18 personal interviews with local policy makers representing municipal authorities. Second, we sent a standardized questionnaire to additional actors involved in regional flood risk management. To identify the most important actors in the sub-catchment area, we analyzed official project documents (e.g., technical reports, meeting minutes) and spoke with multiple experts in flood risk management (following the decisional and reputational approaches; see Knoke 1993). In total, we selected 82 federal, cantonal, and municipal administrative agencies, regional associations, nature conservation organizations, leisure clubs, economic or infrastructure companies, engineering offices, and scientific institutions involved in regional flood risk management (Table 1). The response rate of the standardized questionnaire was 83% (*n* = 68 of 82). We are aware of our small sample and wish to emphasize that the aim of our study is learning from regional flood risk management rather than generalizing our findings.

**Table 1:** Number of survey responses per actor type

Actor type	Number of responses
Federal agency	4
Cantonal agency	11
Municipal agency	18
Regional association	8
Nature conservation organization	7
Leisure club	7
Economic/infrastructure company	6
Engineering office	4
Scientific institutions	3
Total	68

### Operationalization of variables

The first variable *flood exposure* builds on a combined set of data describing recorded flood events and spatial data on affected buildings and residents (Röthlisberger et al. 2017). We considered all documented flood events from 1997 to 2016 along the Aare River between Thun and Bern recorded in the disaster register provided by the Canton Bern. To assess flood exposure, we used GIS tools to first ascribe point-referenced population census data (Federal Statistical Office (FSO) 2018) to spatially compliant building footprint data (Federal Office of Topography (swisstopo) 2018). Next, using geometric intersection analysis, we tested the building footprints for overlaps with the recorded flood areas. In a final step, we aggregated the total number of exposed buildings and residents per municipality (*absolute exposure)*. The absolute exposure of buildings and residents represents a crucial number used to calculate the cost-benefit of measures, since resources are invested in areas where the costs of measures relative to the benefit in terms of flood risk reduction is optimized (Röthlisberger et al. 2017). We analyze flood exposure considering geographical characteristics (e.g., altitude, distance to riverbanks) and also acknowledge exposure as partially a function of past experiences with flood risk management and specific policies.

For the analysis of local governments’ flood exposure in our sub-catchment area, we use the absolute number of exposed residents in the 18 municipalities (for further operationalization, see Table 4 in Appendix 2). The higher the number of exposed residents in a municipality, the higher its flood exposure. Note that this spatial variable is only available for the 18 municipalities^2^ and not for the other (non-spatial) actors.

The second variable *flood risk perception* builds on data from a survey question on actors’ perception of flood risk management trends. In the two sub-questions, we asked actors (1) whether they believe, for their sub-catchment area, that the risk of damage caused by floods is low with the existing flood risk management measures in place and (2) whether they deem the sub-catchment’s population insufficiently prepared for potential future flooding (for the exact wording of the survey sub-questions and further operationalization, see Tables 5 and 6 in Appendix 2). We measured actors’ awareness of flood risks as well as their preparedness for potential future floods in their sub-catchment area. The actors rated the two sub-questions on a 4-point Likert scale ranging from strong agreement to strong disagreement (“fully agree” = value 1; “mostly agree” = value 2; “mostly disagree” = value 3; “fully disagree” = value 4). Based on this data, we created an additive index for flood risk perception. This index ranges from weak perception, with value 2 (e.g., both statements “fully agree”), to strong perception, with value 8 (e.g., both statements “fully disagree”). Finally, we transformed the index into a normalized range [0, 1]. Thus, our index portrays actors as having high flood risk perception when they perceive a high risk of damage caused by potential flooding and simultaneously believe that the population is well prepared for this high risk. Likewise, the index displays actors as having low flood risk perception when they perceive low risk of damage caused by potential flooding combined with population’s low preparedness. Thus, high flood risk perception shows, first, that actors recognize the problem of potential flooding in their sub-catchment area and, second, that they actively address flood risks by preparing the population to it. Our flood risk perception index therefore not only includes peoples’ awareness but also their behavior related to flood risks.

The third variable *policy preferences* builds on data from a policy preferences question in our survey. In a list of statements, we compared two different measures against each other (e.g., dam vs. river widening) and asked actors for each measure to evaluate their preference in comparison with the other measure (for the exact wording of the survey sub-questions, see Table 8 in Appendix 2). The actors rated the contrasted measures on a 4-point Likert scale from strong preference for one measure to strong preference for the other measure (e.g., “prefer option one fully” = value 2; “prefer option one mostly” = value 1; “prefer option two mostly” = value -1; “prefer option two fully” = value -2). As such, the actors indicated for each measure a degree of preference from weak to strong. Following the literature (Hegger et al. 2014; Niven and Bardsley 2013), each proposed measure we contrasted can be assigned to one of the four categories of infrastructure, spatial planning, ecological river restoration, and information (for the assignment of specific measures to categories, see Table 7 in Appendix 2). Based on this data, we constructed an index measuring actors’ mean preferences for each of the four categories of measures. Finally, we transformed the index into a normalized range [0, 1]. The higher the value per category, the stronger the actors’ preferences for these measures.

### Methods

To study the three key variables’ interplay, we combined a spatial approach with correlation analysis. For the spatial actors, i.e., the 18 municipalities in the sub-catchment area, we calculated their degree of flood exposure, flood risk perception, and policy preferences. This approach informs us descriptively about the match or mismatch between local governments’ degrees of flood exposure and flood risk perception with their related preferences for flood risk management measures. For the non-spatial actors, i.e., the 50 remaining actors (see Table 1), this procedure is not possible: they have no assignable area (e.g., scientific institution), are not necessarily located in the investigated sub-catchment area (e.g., federal and cantonal agencies), and have thus no flood exposure values. In addition, we computed for all (spatial and non-spatial) actors the relationship between the three/two variables using the Spearman’s rank-order correlation and Cronbach’s alpha. Due to the small sample and its implications for the reliability of the correlation analyses, we calculated various correlation coefficients with different operationalization options of the two variables flood exposure and flood risk perception (for further information, see Tables 4 and 6 in Appendix 2). All correlation coefficients reveal similar results. We additionally included substantial case knowledge to strengthen our results.

## Results

### Univariate analysis

Table 2 displays municipalities’ flood exposure, flood risk perception, and policy preferences for infrastructure, spatial planning, ecological river restoration, and information measures.

**Table 2:** Municipalities’ flood exposure, flood risk perception, and policy preferences

	Flood exposure	Flood risk perception	Preferences infrastructure	Preferences sp. planning	Preferences ecological	Preferences information
Allmendingen	0	0.50	1.00	0.33	0.33	0.83
Belp	302	0.50	0.73	0.22	0.22	0.50
Bern	4036	0.83	0.25	0.67	0.83	0.83
Gerzensee	8	0.33	0.60	0.67	0.75	0.50
Heimberg	0	0.50	0.20	0.56	0.67	0.67
Jaberg	4	0.50	0.40	0.44	0.67	0.67
Kehrsatz	147	0.33	0.47	0.50	0.67	0.83
Kiesen	183	0.50	0.27	0.56	0.92	0.83
Kirchdorf	46	0.50	0.20	0.44	0.75	0.33
Köniz	350	1.00	0.20	1.00	0.92	1.00
Münsingen	468	0.67	0.47	0.56	0.75	0.83
Muri	61	0.67	0.47	0.67	0.67	0.67
Rubigen	363	0.50	0.33	0.44	0.58	0.83
Steffisburg	157	0.75	0.25	0.56	0.67	0.67
Thun	3575	0.50	0.33	0.33	0.67	0.83
Uetendorf	11	0.67	0.33	0.44	0.67	0.67
Uttigen	10	0.50	0.67	0.11	0.67	0.50
Wichtrach	475	0.50	0.40	0.56	0.67	0.83

*Note*: The table shows absolute values for flood exposure and normalized values on a [0, 1] scale for flood risk perception and policy preferences

Considering flood exposure, half of the municipalities have not been affected by floods at all or only very little (0–100 exposed inhabitants) in the last 20 years. Three municipalities have been slightly exposed to floods with 100–200 affected inhabitants (Kehrsatz, Kiesen, Steffisburg). Five municipalities have been moderately exposed with 300–500 affected inhabitants (Belp, Köniz, Münsingen, Rubigen, Wichtrach). The two most exposed municipalities in the sub-catchment area have been the cities Thun with 3757 and Bern with 4036 affected inhabitants.

Concerning flood risk perception, the values indicate that the majority of the municipalities have moderate to strong flood risk perception. With the exception of two municipalities (Gerzensee, Kehrsatz), flood risk perception lies between 50 and 100%, signifying that almost all municipalities perceive flood risks on their territories and/or in the sub-catchment area.

Comparing policy preferences across the four categories of measures, we notice that the majority of the 18 municipalities prefer spatial planning, ecological river restoration, and information measures to infrastructure measures. Only four municipalities (Allmendingen, Belp, Gerzensee, Uttigen) show moderate to strong preferences for infrastructure measures (i.e., between 50 and 100%), while the other 14 municipalities have rather weak preferences for this category of measure. Ten of the 18 municipalities show moderate to strong preferences for spatial planning tools. Finally, 14 out of 18 municipalities show moderate to strong preferences for information, and 16 municipalities have moderate to strong preferences for ecological river restoration measures.

We find manifold arguments to help explain these results in the context of the sub-catchment area: First, municipalities’ flood exposure values are influenced by geographical factors (Boon 2016). The majority of the municipalities built their village centers with residential zones and important infrastructures (e.g., drinking water wells, roads, and bridges) at some distance or altitude from the riverbanks of the Aare (see also Löschner et al. 2017). These areas are either well protected by agricultural land, forests, or industrial zones or located on a hill or a plateau above the river level. Most municipalities’ inhabitants are therefore rarely affected by floods from the Aare. In contrast, some residential zones and important infrastructures in Thun and Bern, the two municipalities with the highest observed exposure, are located close to the riverbanks of the Aare and are therefore less protected. In such densely populated areas, residential zones often extend to the riverbanks for reasons of space, i.e., urbanization processes also expand into flood-prone areas (Kundzewicz et al. 2014), and aesthetics, i.e., people like pretty views and the exclusiveness of living close to a river (Kron et al. 2019). Furthermore, in all moderate to high exposed municipalities, tributary waters in the sub-catchment area of the Aare are responsible for their increased exposure values. We find one or several tributary waters in each of their territories (e.g., Gürbe in Belp and Kehrsatz). In the case that the Aare floods, the tributaries are also more likely to flood and thus might cause additional flood exposure.

Second, and in line with flood exposure results, we notice that municipalities with residential zones and important infrastructures located close to the Aare or any other waterbody in the sub-catchment area tend to perceive moderate to high flood risks. Despite the assumption that people living in flood-prone areas are often unaware of flood risks or simply ignore them (Kron et al. 2019; Kundzewicz et al. 2018a), the surveyed municipalities seem to hold awareness of the possibility of floods and live consciously with these flood risks, i.e., are willing to behave accordingly in case of flooding (corresponding to the principle “risk taker pays”; Kundzewicz 1999). One factor contributing to such perception might be municipalities’ experience with past flood events affecting their territories, whether from the Aare or other waterbodies. With repeated experiences of flooding, municipalities learn to accept the risk and increase their knowledge of particularly exposed inhabitants, buildings, and infrastructures. The majority of the municipalities with increased levels of flood risk perception have experienced severe damages and losses from several major flood events recently, namely in 1999, 2005, and 2007. Experiencing such heavy flood events and confronting losses is said to awaken people and engender—at least for a limited time—a heightened awareness of flood risks (Kellens et al. 2011; Wachinger et al. 2013). Thus, the surveyed municipalities were learning from these past events and adapting their flood risk awareness accordingly. It also seems that local people’s flood memory is not in danger of fading (see Kundzewicz and Takeuchi 1999), since nearly every year at least minor flood events occur in the studied sub-catchment area (see Fig. 2 in Appendix 1).

Third, policy preferences results are surprising: Municipalities preferring infrastructure measures to protect their population are either situated at some distance or altitude from the riverbanks of the Aare or have important infrastructures located close to the Aare or another waterbody (e.g., regional airport in Belp). Additionally, most of these municipalities have only modest experiences with past flood events. Therefore, implementing the widely established and well-known infrastructure measures, mainly at tributary waters on their territories, seems to be the simplest and most reliable way to address flood risks for those municipalities. In contrast, municipalities preferring spatial planning, ecological river restoration, and information have experienced or perceive that infrastructure measures are not sufficient to protect their population from flood risks and realize that absolute flood resistance is not possible (see Kundzewicz and Takeuchi 1999). These municipalities are aware that preventive measures and keeping people away from the destructive waters are equally essential strategies to reduce people’s exposure to flood risks (see Kundzewicz et al. 2018a). They thus seem to perceive non-structural measures as complementary measures for managing the residual risk and preventing an increase in potential damage. In line with recent literature (Hegger et al. 2016), the surveyed municipalities prefer a combination of structural and non-structural measures to enhance their flood preparedness and effective flood responses. In particular, municipalities’ strong preferences for spatial planning tools and ecological river restoration are remarkable in our densely populated sub-catchment area, since there is little room for such often spacious measures (see Kousky et al. 2013). However, their strong preferences may be explained by municipalities’ ulterior motive of implementing these measures somewhere else in the sub-catchment area to compensate for infrastructure measures enacted in their municipalities.

### Correlation analysis

Table 3 shows the Spearman’s rank-order correlation results, including municipalities and the additional surveyed actors involved in flood risk management.^3^ We find that municipalities’ flood risk perception is moderately negatively correlated with their preferences for infrastructure measures and moderately positively correlated with their preferences for spatial planning measures. This trend is confirmed by all actors’ correlation coefficients, adding a moderate positive correlation of flood risk perception with their preferences for ecological river restoration. Municipalities’ flood exposure is not significantly linked with their flood risk perception (corr. coeff. 0.37, *p* > 0.1); however, it shows a moderate positive correlation with their preferences for information tools. Results are consistent when comparing Spearman’s rank-order correlation to Cronbach’s alpha (0.73, CI: 0.65, 0.82; for further information, see Table 9 in Appendix 3).

**Table 3:** Correlation coefficients of flood exposure and flood risk perception to policy preferences

	Flood exposure (municipalities; *n* = 18)	Flood risk perception (municipalities; *n* = 18)	Flood risk perception (all actors; *n* = 68)
Infrastructure	− 0.22	− 0.43*	− 0.40***
Spatial planning	0.21	0.41*	0.39***
Ecological river restoration	0.24	0.30	0.45***
Information	0.55**	0.28	0.05

*Note*: All correlations are Spearman’s rank-order

*** *p* < 0.01; ** *p* < 0.05; * *p* < 0.1

Following our correlation results, we have to reject our second and third hypotheses: municipalities’ high flood exposure and flood risk perception in fact enhance preferences not mainly for structural but rather for diversified measures. In contrast, we can neither corroborate nor reject our first hypothesis, because we find no link between municipalities’ flood exposure and flood risk perception. Nevertheless, our results still provide some important insights into decision-making mechanisms related to flood risks that we wish to contextualize in the following section.

### Local context

Embedding our results in the surveyed sub-catchment area, we emphasize two points: First, flood-aware municipalities prefer to not only rely on infrastructure measures but to also implement non-structural measures to reduce their flood risks. These preferences align with the Federal Act on Hydraulic Engineering (Federal Act of 21 June 1991 on Hydraulic Engineering), which prioritizes non-structural measures, and in particular spatial planning measures, to reduce flood risks, and allows for structural measures to be implemented only in the case of insufficient protection by non-structural measures (article 3, paragraphs 1 and 2). Despite the clear legal framework, municipalities’ preferred non-structural measures are implemented in only a few cases, mainly complementing the well-known infrastructure measures (see Koks et al. 2014; de Moel et al. 2013). Municipalities’ preferences and the implemented flood risk management measures in the studied sub-catchment area thus do not correspond. This mismatch could be due to various developments in the sub-catchment area.

Under guidance of the Canton Bern, a participatory approach brought together a wide range of flood-affected actors from different policy sectors (see Table 1) to design an integrated flood risk management approach. However, flood risk management converged with other interests such as protection of drinking water wells and the extensive cultivation of forest and agricultural land. These conflicting interests led some actors to block the process for several years, which resulted in a halt of the project. The intensely negotiated distribution formula of costs between municipalities according to the solidarity perspective (see Kundzewicz et al. 2018a) became obsolete. Today, in the sub-catchment area, only the formerly planned measures that do not conflict with any other interests and sectors, or at minimum offer a compromise between the different interests and sectors, are being implemented. These are, however, often upgraded or new structural measures complemented by some soft non-structural measures, which do not align with municipalities’ preferences. To avoid further conflict, municipalities therefore continue to rely on similar structural measures to those that they have previously implemented and which proved effective during past flood events (according to the concept of path dependency). The final selection and implementation of flood risk management measures in the surveyed sub-catchment area is hence the result of negotiations between powerful actors in the political flood process and municipalities’ experiences with past measures, rather than an adherence to facts, such as increasing exposure and vulnerability of the local population.

Second, flood risk perception is a key factor determining actors’ preferences for flood risk management measures. Local citizens’ increased flood risk perception grants the municipalities the legitimacy to implement measures that reduce flood risks (see Botzen et al. 2009). However, flood risk perception is not only a necessary condition for municipalities to implement measures but also affects municipalities’ preferences for the specific design of their flood risk management portfolio. Our case study shows that high flood risk perception shapes preferences for diversified flood risk management portfolios, i.e., flood-aware municipalities prefer combined integrative strategies and preventive measures, surpassing the traditional structural measures implemented in most surveyed municipalities. We learn from our case study that for flood risk management it is essential to maintain people’s high flood awareness, mainly between two flood events when flood memories possibly fade (Kundzewicz 1999). While influencing flood risk perception proves difficult (Kundzewicz et al. 2018a), some experiences in our sub-catchment area illustrate potential ways forward.

One option for promoting flood risk perception entails applying adapted communication strategies and developing intuitive visual materials about local flood risks for lay people (for an example, see also Kundzewicz et al. 2018a). In all surveyed municipalities, flood hazard maps are accessible online, and most municipalities communicated regularly about how to potentially reduce flood risks during the integrated flood risk management approach using various strategies, such as articles in the local newspaper, information boards on-site, public site inspections, and assemblies. Another option is to enhance the general public knowledge about flood risks and flood risk management in educational campaigns via schools, community context, expert communication, mass media, etc. This option would, for instance, include simple (visual) explications of multiple mechanisms causing floods and how these mechanisms can be linked to human activities. Such educational activities could increase the public’s sensitivity to flood-related information and news and foster acceptance of and support for flood policies (Otto-Banaszak et al. 2011; Otto et al. 2020). In our sub-catchment area, the project leader Canton Bern occasionally applied this option by giving public presentations in the surveyed municipalities or publishing interviews with the cantonal councilor responsible for flood risk management. A last option to increase flood awareness proposed here entails fostering open debate and participatory approaches. This option contributes to deliberation, justice, acceptability, and legitimacy of measures since it opens the discussion up to what is understood as desirable and how and to what extent different measures might reduce flood risks (Alexander et al. 2018). From a normative perspective, the consideration of citizens’ opinions might provide a way for them to feel more affected and to transfer responsibility onto them. However, as seen in our sub-catchment area, participatory approaches can also provoke conflicts, the slowdown of processes, or, in the worst-case scenario, a project coming to a halt altogether.

For all three awareness-raising strategies mentioned above, in line with Kundzewicz et al. (2020), we consider it important to frame flood risks and flood risk management messages positively to increase people’s risk perception, rather than to create an “atmosfear” of risk, damage, and fear. In summary, to maintain people’s high flood risk perception, predominantly during long periods without flooding, engendering an affected population could prove helpful, i.e., to force citizens to handle flood risks and flood risk management, as shown in our surveyed sub-catchment area. Provoking affectedness can be achieved either through simple and easily accessible scientific data or through the more emotionally centered approach of including actors in normative debates. It should be noted that the latter strategy requires policy makers to make considerable efforts for citizens’ routine involvement. When confronted with such awareness-raising strategies, however, an interviewed municipal representative in our sub-catchment area stated that the only lasting solution for affecting citizens and maintaining their high awareness would likely comprise the natural effect of major flood events occurring in regular intervals.

## Discussion and policy implications

Continuous urban developments in many flood-prone areas in Europe increase the number of people exposed to flood risks (Nicholls et al. 2008; Kron et al. 2019; Kundzewicz et al. 2014), but their flood risk perception and policy preferences for measures to reduce these risks do not follow this trend. As such, designing effective flood risk management and understanding the interplay between flood exposure, flood risk perception, and policy preferences are crucial, particularly for local governments. Based on the novel combination of risk analysis and public policy data and methods, we analyzed the three variables in the ideal context of 18 Swiss municipalities in a sub-catchment area of the Aare River. Results illustrate that local governments perceiving high flood risks tend to prefer non-structural measures, such as spatial planning and ecological river restoration, to infrastructure measures.

In line with several recent studies in the context of Switzerland (e.g., Buchecker et al. 2016), our results support the notion that the widespread infrastructure measures are no longer the sole and undisputable policy solution to address flood risks. Non-structural measures are becoming more vital and universally implemented. However, even though subsidized by the federal government, current spatial planning and ecological tools are primarily understood as complementary rather than stand-alone measures. This trend in Swiss flood risk management needs to be embedded in the Swiss institutional context. Cantons holding the responsibilities in Swiss flood risk management often conflict with other policy sectors (e.g., agriculture) and actor groups (e.g., NGOs, private landowners) (Zaugg Stern 2006). The final selection and implementation of flood risk management measures is therefore the result of political power play and conflictive negotiations between ideologically different actors (see Bressers and O’Toole 2005). Thus, instead of local governments’ preferred options, second-choice measures are often implemented as a compromise between varying interests (see Knill and Lenschow 2005). At the same time, local governments tend to maintain the established infrastructure measures that function well and have known outcomes (see Peters et al. 2005; Pierson 2000). Therefore, experiences with past flood risk management measures as well as the institutional framework characterize today’s Swiss flood risk management.

Going beyond the case of Switzerland, our study illustrates that studying flood risk management is contingent upon the local context, which has its own structures, political agenda, or opinion-forming and decision-making mechanisms. It is therefore vital to consider the institutions and arenas in which objectives and principles of flood risk management are negotiated (Zaugg Stern 2006). The regional and local institutional, socio-political, and economic environment is of crucial importance in explaining various flood risk management portfolios (Bubeck et al. 2017; Otto-Banaszak et al. 2011). At the same time, however, actors’ flood risk perception generally matters for flood risk preparedness regardless of local differences in flood exposure and policy preferences. Several studies found a positive correlation between actors’ flood risk perception and their preventive behaviors or disaster preparedness (e.g., Miceli et al. 2008). Our results support the importance of flood risk perception and take this conclusion one step further: flood risk perception not only explains whether or not actors implement flood risk management measures but also affects the specific *design* of flood risk management portfolios. Thus, high flood risk perception may help to achieve diversification towards different combined flood risk management strategies and measures. As a result, local governments in flood-prone areas may actively try to increase their population’s awareness of flood risks, for instance, with lay people–adapted communication and visual materials on local flood risks, general flood education, or participatory approaches (see Alexander et al. 2018; Kundzewicz et al. 2018a; Otto et al. 2020; Otto-Banaszak et al. 2011). Such strategies can stimulate actors’ preferences for the implementation of specific diversified policy portfolios including more preventive or integrated measures.

Our study undoubtedly has several limitations. We collected data for one sub-catchment area and surveyed a small number of actors. Our findings are therefore context-sensitive and call for further research. Future studies should concentrate on a greater geographic area or expand to compare several (sub-)catchment areas in different regions with diverging institutional, socio-political, economic, and geographic contexts—as well as with heterogeneous experiences in flood risk management. Another point worth noting is our strong focus on actors’ risk awareness as an important factor influencing flood risk management strategies. Risk awareness constitutes a passive approach to flood risk management, and the design, selection, and implementation of more diversified non-structural measures often requires actors’ active participation. Future work should analyze actors’ willingness to actively participate in flood risk management processes, contrasting active participation with passive perception and its significance for flood risk management.

## Appendix 1. Case study

Figure 1 illustrates the study area of the Aare River with its 18 municipalities and multiple waterbodies. This sub-catchment area of the Aare River between the cities Thun and Bern is part of the larger Aare catchment in the Canton Bern.

Figure 2 illustrates historical flood records in our study area from 1995 to 2017. For each year, the figure shows the total number of floods (several floods occurring throughout the sub-catchment area at the same time are part of the same flood event) and, thereof, the ones having caused at least one damage either to people, animals, properties, roads, railway lines, infrastructure, or forest and agricultural land. The data used for this figure is provided in the disaster register by the Canton Bern (Forest and Natural Hazard Office Canton Bern (KAWA)).

## Appendix 2. Operationalization of variables

### Appendix 2.1. Operationalization of flood exposure

We considered several different operationalization options for our variable flood exposure including the absolute values and the ratio for modelled and recorded flood exposure, which can be seen in Table 4. The operationalization we finally used for our analysis is option (8) (marked in bold).

**Table 4:** Operationalization options of variable flood exposure

Option	Operationalization
Flood exposure (1)	Ratio of exposed buildings in the total number of buildings in a municipality (modelled exposure, according to hazard map)
Flood exposure (2)	Ratio of exposed persons in the total population of a municipality (modelled exposure, according to hazard map)
Flood exposure (3)	Absolute number of exposed buildings in a municipality (modelled exposure, according to hazard map)
Flood exposure (4)	Absolute number of exposed persons in a municipality (modelled exposure, according to hazard map)
Flood exposure (5)	Ratio of exposed buildings in the total number of buildings in a municipality (recorded exposure, overlap with flooded areas according to the disaster register by the Canton Bern)
Flood exposure (6)	Ratio of exposed persons in the total population of a municipality (recorded exposure, overlap with flooded areas according to the disaster register by the Canton Bern)
Flood exposure (7)	Absolute number of exposed buildings in a municipality (recorded exposure, overlap with flooded areas according to the disaster register by the Canton Bern)
Flood exposure (8)	Absolute number of exposed persons in a municipality (recorded exposure, overlap with flooded areas according to the disaster register by the Canton Bern)

### Appendix 2.2. Operationalization of flood risk perception

We considered several different survey sub-questions and various operationalization options for our variable flood risk perception. The survey sub-questions can be seen in Table 5 and the operationalization options in Table 6. For our analysis, we finally used the sub-questions (4) and (6) and combined them in an additive index, the operationalization option (8) (all marked in bold).

**Table 5:** Survey sub-questions considered for variable flood risk perception

Survey question/statement	Response options	Operationalization index
1) The number of flood events in the area along the Aare between Thun and Bern has increased over the last 20 years 2) The extent (river runoff) of flood events in the area along the Aare between Thun and Bern has increased over the last 20 years 3) The damage caused by flood events in the area along the Aare between Thun and Bern has increased over the last 20 years 4) The risk of damage caused by flood events in the area along the Aare between Thun and Bern is low with the existing protection measures in place 5) The population in the area along the Aare between Thun and Bern is well informed about regional flood hazards and flood-prone areas 6) The population in the area along the Aare between Thun and Bern is insufficiently prepared for potential further flood events 7) The risk of potential further flood events in the area along the Aare between Thun and Bern is causing uncertainty among the population 8) Organizations involved in flood risk management in the area along the Aare between Thun and Bern should cooperate closer in the future to reduce uncertainties regarding flood risks 9) The unknown effects of damage caused by potential further flood events in the area along the Aare between Thun and Bern result in few preventive measures being taken 10) Organizations involved in flood risk management in the area along the Aare between Thun and Bern need to be better and more regularly informed about flood hazards by the responsible agencies	Fully agree; mostly agree; mostly disagree; fully disagree	Additive index of the two statements (4) and (6) with a normalized scale from 0 to 1

**Table 6:** Operationalization options of variable flood risk perception

Option	Operationalization
Flood risk perception (1)	Survey sub-questions 1–3 (see Table 5)
Flood risk perception (2)	Survey sub-questions 1–4 (see Table 5)
Flood risk perception (3)	Survey sub-questions 1–7 (see Table 5)
Flood risk perception (4)	Survey sub-questions 1–10 (see Table 5)
Flood risk perception (5)	Survey sub-questions 1–3, 6, 7 (see Table 5)
Flood risk perception (6)	Survey sub-questions 1–3, 8–10 (see Table 5)
Flood risk perception (7)	Survey sub-questions 4, 6, 7 (see Table 4)
Flood risk perception (8)	Survey sub-questions 4, 6 (see Table 5)
Flood risk perception (9)	Survey sub-questions 4, 6; sub-question 6 is coded reversed (see Table 5)
Flood risk perception (10)	Survey sub-question 4 (see Table 5)

### Appendix 2.3. Operationalization of policy preferences

The specific flood risk management measures belonging to one of the four categories of infrastructure, spatial planning, ecological river restoration, and information can be seen in Table 7.

**Table 7:** Categories and specific flood risk management measures

Categories of flood risk management measures	Examples of specific flood risk management measures
Infrastructure	Flood protection dam; hard bank reinforcement; river regulation; river bed stabilization
Spatial planning	Preventive construction ban/restriction; flood retention area; drainage corridor; distance to waters
Ecological river restoration	River widening; natural and dynamic river landscape; conservation of floodplain areas; new space for waterbodies
Information	Flood protection exercise/training; warning systems; emergency plans

The exact operationalization of our variable policy preferences with the corresponding sub-question in the survey can be seen in Table 8.

**Table 8:** Operationalization of variable policy preferences

Survey question / Statement			Response options	Operationalizationindex
Please indicate your organization’s references for the following opposing options of measures:			Prefer option 1 fully;Prefer option 1 mostly;Prefer option 2 mostly;Prefer option 2 fully	Mean index of thestatements percategory(infrastructure, spatialplanning, ecologicalriver restoration,information) with anormalized scale from[0, 1]
	Option 1	Option 2		
1	infrastructure measure	spatial planning measure		
2	flood protection dam	river widening		
3	flood retention area	hard bank reinforcement		
4	hard bank reinforcements	natural river landscape		
5	river bed stabilization	natural river landscape		
6	preventive construction ban	flood protection dam		
7	flood retention area	river regulation		
8	ecological river restoration	infrastructure measure		
9	infrastructure measure	flood protection exercise		
10	other measures	infrastructure measure		
11	infrastructure measure	conservation of floodplainareas		
12	flood protection dam	more space for waterbodies		
13	relocation of groundwaterwells	infrastructure measure		
14	warning systems	other measures		

## Appendix 3. Correlation analysis

### Appendix 3.1. Summary statistics

Table 9 contains summary statistics of the three variables: flood exposure, flood risk perception, and policy preferences.

**Table 9:** Summary table of flood exposure, flood risk perception, and policy preferences

	Mean	SD	Min	Median	Max	Alpha	Cases	n.a.
Flood exposure	0.04	0.05	0.00	0.03	0.20	---	18	0
Flood risk perception	0.57	0.14	0.17	0.50	1.00	0.71	82	22
Infrastructure	0.35	0.23	0.00	0.33	1.00	0.59	82	16
Spatial planning	0.55	0.23	0.00	0.56	1.00	0.63	82	17
Ecological river restoration	0.73	0.22	0.00	0.75	1.00	0.59	82	18
Information	0.76	0.19	0.17	0.83	1.00	0.82	82	18

Cronbach’s alpha illustrates the reliability of the variables’ indices for the case of each item being removed one by one. There is no Cronbach’s alpha for flood exposure, since this variable is not based on a Likert scale

### Appendix 3.2. Additional correlation coefficients

We calculated several additional correlation coefficients with a different operationalization of our variables flood exposure and flood risk perception. Table 10 shows the additional correlation coefficients for flood exposure and flood risk perception, Table 11 for flood exposure and policy preferences, and Table 12 for flood risk perception and policy preferences. These additional correlation coefficients are included to show the robustness of our correlation results for our sub-catchment area. Calculating systematically additional correlation coefficients gives us very similar results compared with our original correlation coefficients. However, we are fully aware that due to the small sample size, these correlation results only apply to the sub-regional context and do not claim generalization for larger areas, such as the Canton Bern, or Switzerland at large.

**Table 10:** Additional correlation coefficients flood exposure—flood risk perception

		Flood exposure (1)	Flood exposure (2)	Flood exposure (3)	Flood exposure (4)	Flood exposure (5)	Flood exposure (6)	Flood exposure (7)	Original flood exposure (8)
Municipalities (n = 18)	Flood risk perception (1)	0.01	− 0.13	0.21	0.13	0.34	0.38	0.30	0.33
	Flood risk perception (2)	− 0.05	− 0.17	0.28	0.17	0.18	0.23	0.32	0.36
	Flood risk perception (3)	− 0.05	− 0.18	0.27	0.18	0.29	0.35	0.39	0.44*
	Flood risk perception (4)	− 0.10	− 0.20	0.28	0.18	0.21	0.25	0.37	0.42*
	Flood risk perception (5)	0.00	− 0.16	0.20	0.12	0.43*	0.48**	0.38	0.41*
	Flood risk perception (6)	− 0.03	− 0.17	0.24	0.16	0.25	0.26	0.28	0.30
	Flood risk perception (7)	− 0.40	− 0.40*	0.26	0.24	− 0.08	− 0.12	0.43*	0.52**
	Original flood risk perception (8)	− 0.28	− 0.25	0.30	0.33	− 0.26	− 0.23	0.27	0.37
	Flood risk perception (9)	− 0.18	− 0.13	0.26	0.18	− 0.28	− 0.34	0.19	0.19
	Flood risk perception (10)	− 0.27	− 0.23	0.33	0.29	− 0.29	− 0.32	0.29	0.33

*Note*: All correlations are Spearman’s rank-order

*** *p* < 0.01; ** *p* < 0.05; * *p* < 0.1

**Table 11:** Additional correlation coefficients flood exposure—policy preferences

		Infrastructure	Spatial planning	Ecological	Information
Municipalities (n = 18)	Flood exposure (1)	− 0.04	− 0.04	0.10	0.03
	Flood exposure (2)	− 0.01	− 0.01	0.12	− 0.04
	Flood exposure (3)	− 0.19	0.05	0.12	0.20
	Flood exposure (4)	− 0.34	0.17	0.22	0.26
	Flood exposure (5)	− 0.04	− 0.11	0.09	0.28
	Flood exposure (6)	− 0.16	− 0.11	0.20	0.30
	Flood exposure (7)	− 0.20	0.18	0.22	0.48**
	Original Flood exposure (8)	− 0.22	0.21	0.24	0.55**

*Note*: All correlations are Spearman’s rank-order

*** *p* < 0.01; ** *p* < 0.05; * *p* < 0.1

**Table 12:** Additional correlation coefficients flood risk perception—policy preferences

		Infrastructure	Spatial planning	Ecological	Information
Municipalities (n = 18)	Flood risk perception (1)	− 0.10	− 0.36	− 0.03	− 0.03
	Flood risk perception (2)	− 0.15	− 0.20	0.03	− 0.03
	Flood risk perception (3)	− 0.17	− 0.17	0.07	0.11
	Flood risk perception (4)	− 0.15	− 0.15	0.04	− 0.01
	Flood risk perception (5)	− 0.11	− 0.29	0.02	0.11
	Flood risk perception (6)	− 0.05	− 0.36	− 0.10	− 0.15
	Flood risk perception (7)	− 0.12	0.39	0.09	0.46*
	Original flood risk perception (8)	− 0.43*	0.41*	0.30	0.28
	Flood risk perception (9)	0.01	0.33	0.09	− 0.09
	Flood risk perception (10)	− 0.23	0.39	0.21	0.11
All actors (n = 68)	Flood risk perception (1)	− 0.37***	0.10	0.29**	0.09
	Flood risk perception (2)	− 0.39***	0.17	0.36***	0.13
	Flood risk perception (3)	− 0.35***	0.11	0.38***	0.17
	Flood risk perception (4)	− 0.37***	0.16	0.43***	0.20
	Flood risk perception (5)	− 0.29**	0.06	0.29**	0.12
	Flood risk perception (6)	− 0.38***	0.15	0.35***	0.12
	Flood risk perception (7)	− 0.27**	0.29**	0.42***	0.10
	Original flood risk perception (8)	− 0.40***	0.39***	0.45***	0.05
	Flood risk perception (9)	− 0.29**	0.20**	0.23*	0.06
	Flood risk perception (10)	− 0.34***	0.28***	0.32**	0.09

*Note*: All correlations are Spearman’s rank-order

*** *p* < 0.01; ** *p* < 0.05; * *p* < 0.1

## Footnotes

1 Political actors are individuals or groups of individuals with direct or indirect government or non-government affiliations who seek to influence the outcome of a policy process (Weible and Ingold 2018). Political actors can include representatives from government agencies, interest groups, NGOs, industry, or scientific institutions.

2 The general term “municipality” refers to both municipal authorities and residents, the most important local actors in this article.

3 Further correlation coefficients achieve similar results and can be found in Tables 10, 11, and 12 in Appendix 3. Our mathematical calculations have to be taken with caution due to the limited explanatory power of the small sample. However, we wish to emphasize that the aim of this study is to shed light on and learn from regional flood risk management rather than to generalize our results.

## References

[CR1] Alexander M, Doorn N, Priest S (2018). Bridging the legitimacy gap - translating theory into practical signposts for legitimate flood risk governance. Reg Environ Chang.

[CR2] Baron N, Petersen LK (2015). Climate change or variable weather: rethinking Danish homeowners’ perceptions of floods and climate. Reg Environ Chang.

[CR3] Bezzola GR, Hegg C (2007) Event analysis of the flooding 2005, part 1 - processes, damages and first classification [Ereignisanalyse Hochwasser 2005, Teil 1 – Prozesse, Schäden und erste Einordnung]. https://www.bafu.admin.ch/bafu/de/home/themen/naturgefahren/publikationen-studien/publikationen/ereignisanalyse-hochwasser-2005-prozesse-schaeden-und-erste-einordnungen.html. Accessed 05 Apr 2020

[CR4] Boon HJ (2016). Perceptions of climate change risk in four disaster-impacted rural Australian towns. Reg Environ Chang.

[CR5] Botzen WJW, Aerts JCJH, van den Bergh JCJM (2009). Dependence of flood risk perceptions on socioeconomic and objective risk factors. Water Resour Res.

[CR6] Bressers HTA, O’Toole LJ, Eliadis P, Hill M, Howlett M (2005). Instrument selection and implementation in a networked context. Designing government: from instruments to governance.

[CR7] Bubeck P, Botzen WJW, Aerts JCJH (2012). A review of risk perceptions and other factors that influence flood mitigation behavior. Risk Anal.

[CR8] Bubeck P, Kreibich H, Penning-Rowsell EC, Botzen WJW, de Moel H et al (2017) Explaining differences in flood management approaches in Europe and in the USA - a comparative analysis. J Flood Risk Manag 10(4):436–445. 10.1111/jfr3.12151

[CR9] de Moel H, van Vliet M, Aerts JCJH (2013). Evaluating the effect of flood damage-reducing measures: a case study of the unembanked area of Rotterdam, the Netherlands. Reg Environ Chang.

[CR10] Dermont C, Ingold K, Kammermann L, Stadelmann-Steffen I (2017). Bringing the policy making perspective in: a political science approach to social acceptance. Energy Policy.

[CR11] Driessen PPJ, Hegger DLT, Bakker MHN, van Rijswick H, Kundzewicz ZW (2016) Toward more resilient flood risk governance. Ecol Soc 21(4). 10.5751/ES-08921-210453

[CR12] Driessen PPJ, Hegger DLT, Kundzewicz ZW, van Rijswick HFMW, Crabbé A et al (2018) Governance strategies for improving flood resilience in the face of climate change. Water 10(11):1595. 10.3390/w10111595

[CR13] Federal Act of 21 June 1991 on Hydraulic Engineering

[CR14] Federal Office for the Environment (FOEN) Hydrological data and forecasts. Aare - Bern, Schönau. https://www.hydrodaten.admin.ch/lhg/sdi/hq_studien/hq_statistics/2135hq.pdf. Accessed 05 Apr 2020

[CR15] Federal Office of Topography (swisstopo) (2018) swissTLM3D, The topographic landscape model TLM (of Switzerland). https://shop.swisstopo.admin.ch/en/products/landscape/tlm3D. Accessed 01 Sep 2018

[CR16] Federal Statistical Office (FSO) Buildings and dwellings statistic (Since 2009): factsheet - surveys, Sources. https://www.bfs.admin.ch/bfs/en/home/statistics/construction-housing/surveys/gws2009.html (September 1, 2018). Accessed 01 Sep 2018

[CR17] Forest and Natural Hazard Office Canton Bern (KAWA) Disaster register NGKAT. https://www.geo.apps.be.ch/de/geodaten/suche-nach-geodaten.html?view=sheet&preview=search_list&catalog=geocatalog&type=complete&guid=d7bc5d94-fa01-42d6-aca9-bbcb1437551a. Accessed 03 Jan 2018

[CR18] Gerber J-D, Knoepfel P, Nahrath S, Varone F (2009). Institutional resource regimes: towards sustainability through the combination of property-rights theory and policy analysis. Ecol Econ.

[CR19] Gralepois M, Larrue C, Wiering M, Crabbé A, Tapsell S et al (2016) Is flood defense changing in nature? Shifts in the flood defense strategy in six European countries. Ecol Soc 21(4). 10.5751/ES-08907-210437

[CR20] Griffin RJ, Yang Z, ter Huurne E, Boerner F, Ortiz S et al (2008) After the flood: anger, attribution, and the seeking of information. Sci Commun 29(3):285–315. 10.1177/1075547007312309

[CR21] Hegger DLT, Driessen PPJ, Dieperink C, Wiering M, Raadgever GTT et al (2014) Assessing stability and dynamics in flood risk governance. An empirically illustrated research approach. Water Resour Manag 28(12):4127–4142. 10.1007/s11269-014-0732-x

[CR22] Hegger DLT, Driessen PPJ, Wiering M, van Rijswick HFMW, Kundzewicz ZW et al (2016) Toward more flood resilience: Is a diversification of flood risk management strategies the way forward? Ecol Soc 21(4). 10.5751/ES-08854-210452

[CR23] Howlett M, Eliadis P, Hill M, Howlett M (2005). What is a policy instrument? Tools, mixes, and implementation styles. Designing government: From instruments to governance.

[CR24] Ingold K, Gavilano A, Bynander F, Nohrstedt D (2020). Under what conditions does an extreme event deploy its power? Towards collaborative management in Swiss flood risk management. Collaborative crisis management – inter-organizational approaches to extreme events.

[CR25] Intergovernmental Panel on Climate Change (IPCC) (2007) Fourth Assessment Report. Working Group II: Impacts, Adaptation and Vulnerability. Chapter 3.4.3 Floods and Droughts. https://www.ipcc.ch/publications_and_data/ar4/wg2/en/ch3s3-4-3.html (May 1, 2017). Accessed 01 May 2017

[CR26] Intergovernmental Panel on Climate Change (IPCC) (2012). Managing the risks of extreme events and disasters to advance climate change adaptation. A special report of working groups I and II of the Intergovernmental Panel on Climate Change.

[CR27] Janković V, Schultz DM (2017). Atmosfear: communicating the effects of climate change on extreme weather. Weather Clim Soc.

[CR28] Kellens W, Zaalberg R, Neutens T, Vanneuville W, de Maeyer P (2011). An analysis of the public perception of flood risk on the Belgian coast. Risk Anal.

[CR29] Knill C, Lenschow A (2005). Compliance, competition and communication: different approaches of European governance and their impact on national institutions. J Common Mark Stud.

[CR30] Knoke D (1993). Networks of elite structure and decision making. Sociol Methods Res.

[CR31] Koks EE, de Moel H, Aerts JCJH, Bouwer LM (2014). Effect of spatial adaptation measures on flood risk: study of coastal floods in Belgium. Reg Environ Chang.

[CR32] Koks EE, Jongman B, Husby TG, Botzen WJW (2015). Combining hazard, exposure and social vulnerability to provide lessons for flood risk management. Environ Sci Pol.

[CR33] Kousky C, Olmstead SM, Walls MA, Macauley M (2013). Strategically placing green infrastructure: cost-effective land conservation in the floodplain. Environ Sci Technol.

[CR34] Kron W, Eichner J, Kundzewicz ZW (2019). Reduction of flood risk in Europe – reflections from a reinsurance perspective. J Hydrol.

[CR35] Kundzewicz ZW (1999). Flood protection - sustainability Issues. Hydrol Sci J.

[CR36] Kundzewicz ZW, Takeuchi K (1999). Flood protection and management: quo vadimus?. Hydrol Sci J.

[CR37] Kundzewicz ZW, Kanae S, Seneviratne SI, Handmer J, Nicholls N et al (2014) Flood risk and climate change: global and regional perspectives. Hydrol Sci J 59(1):1–28. 10.1080/02626667.2013.857411

[CR38] Kundzewicz ZW, Hegger DLT, Matczak P, Driessen PPJ (2018). Flood-risk reduction: structural measures and diverse strategies. Proc Natl Acad Sci U S A.

[CR39] Kundzewicz ZW, Pińskwar I, Brakenridge GR (2018). Changes in river flood hazard in Europe: a review. Hydrol Res.

[CR40] Kundzewicz ZW, Matczak P, Otto IM, Otto PE (2020). From “atmosfear” to climate action. Environ Sci Pol.

[CR41] Leiserowitz A (2006). Climate change risk perception and policy preferences: the role of affect, imagery, and values. Clim Chang.

[CR42] Löschner L, Herrnegger M, Apperl B, Senoner T, Seher W et al (2017) Flood risk, climate change and settlement development: a micro-scale assessment of Austrian municipalities. Reg Environ Chang 17(2):311–322. 10.1007/s10113-016-1009-0

[CR43] Messner F, Meyer V (2006) Flood damage, vulnerability and risk perception – challenges for flood damage research. In: Schanze J, Zeman E, Marsalek J (eds) Flood risk management: hazards, vulnerability and mitigation measures. Springer Netherlands, Dordrecht

[CR44] Miceli R, Sotgiu I, Settanni M (2008). Disaster preparedness and perception of flood risk: a study in an alpine valley in Italy. J Environ Psychol.

[CR45] Nicholls RJ, Hanson S, Herweijer C, Patmore N, Hallegatte S et al (2008) Ranking port cities with high exposure and vulnerability to climate extremes. Exposure Estimates. OECD Publishing, Paris

[CR46] Niven RJ, Bardsley DK (2013). Planned retreat as a management response to coastal risk: a case study from the Fleurieu Peninsula, South Australia. Reg Environ Chang.

[CR47] O'Neill E, Brereton F, Shahumyan H, Clinch JP (2016). The impact of perceived flood exposure on flood-risk perception: the role of distance. Risk Anal.

[CR48] Ostrom E (2000) The danger of self-evident truths. PS: Polit Sci Polit 33(1):33–44. 10.2307/420774

[CR49] Otto IM, Wiedermann M, Cremades R, Donges JF, Auer C et al (2020) Human agency in the Anthropocene. Ecol Econ 167:106463. 10.1016/j.ecolecon.2019.106463

[CR50] Otto-Banaszak I, Matczak P, Wesseler J, Wechsung F (2011). Different perceptions of adaptation to climate change: a mental model approach applied to the evidence from expert interviews. Reg Environ Chang.

[CR51] Peters BG, Pierre J, King DS (2005) The politics of path dependency: political conflict in historical institutionalism. J Polit 67(4):1275–1300. 10.1111/j.1468-2508.2005.00360.x

[CR52] Pierson P (2000) Increasing returns, path dependence, and the study of politics // Increasing returns, path dependence, and the study of politics. Am Polit Sci Rev 94(2):251–267. 10.2307/2586011

[CR53] Röthlisberger V, Zischg AP, Keiler M (2017). Identifying spatial clusters of flood exposure to support decision making in risk management. Sci Total Environ.

[CR54] Slovic P (1987). Perception of risk. Science.

[CR55] Slovic P, Finucane ML, Peters E, MacGregor DG (2004). Risk as analysis and risk as feelings: some thoughts about affect, reason, risk, and rationality. Risk Anal.

[CR56] Suter H, Thomi L, Weingartner R, Zischg A (2016). What makes flood control projects successful? [Was macht Hochwasserschutzprojekte erfolgreich?]. Wasser Energie Luft.

[CR57] Tanner A, Árvai J (2018). Perceptions of risk and vulnerability following exposure to a major natural disaster: the Calgary Flood of 2013. Risk Anal.

[CR58] United Nations Office for Disaster Risk Reduction (UNISDR) (2009) 2009 UNISDR Terminology on disaster risk reduction. https://www.unisdr.org/files/7817_UNISDRTerminologyEnglish.pdf. Accessed 5 July 2018

[CR59] Wachinger G, Renn O, Begg C, Kuhlicke C (2013). The risk perception paradox - implications for governance and communication of natural hazards. Risk Anal.

[CR60] Weible CM, Ingold K (2018). Why advocacy coalitions matter and practical insights about them. Policy Polit.

[CR61] Zaugg Stern M (2006) Philosophy shift in hydraulic engineering: on the implementation practice of sustainable flood risk management [Philosophiewandel im schweizerischen Wasserbau: Zur Vollzugspraxis des nachhaltigen Hochwasserschutzes]. Ph.D. Thesis, Schriftenreihe Humangeographie, vol 20. Faculty of Science, University of Zurich, Zurich

[CR62] Buchecker M, Ogasa DM, Maidl E (2016) How well do the wider public accept integrated flood risk management? An empirical study in two Swiss Alpine valleys. Environ Sci Pol 55:309–317. 10.1016/j.envsci.2015.07.021

